# Emerging approaches to redressing multi-level racism and reproductive health disparities

**DOI:** 10.1038/s41746-022-00718-2

**Published:** 2022-11-04

**Authors:** Bethany Golden, Ifeyinwa V. Asiodu, Linda S. Franck, Celestine Yayra Ofori-Parku, Daniel Felipe Martín Suárez-Baquero, Tracy Youngston, Monica R. McLemore

**Affiliations:** 1 Department of Family Health Care Nursing, School of Nursing, University of California, San Francisco, 2 Koret Way, Box 0606, San Francisco, CA 94543 USA; 2 Department of Family Health Care Nursing, University of California, San Francisco, 2 Koret Way, Box 0606, San Francisco, CA 94543 USA

**Keywords:** Health care, Risk factors

## Abstract

This commentary examines the impact of multi-level racism on reproductive health disparities in the United States. Multi-level racism and its impact on reproductive health over the lifespan are described on a societal, community, and individual level. To advance, we recommend using the Remove, Repair, Restructure, Remediate (R4P) approach combined with the Retrofit, Reform, and Reimagine (3R) model to address multiple forms of racism. Emergent policies and actions are identified to proceed towards health equity.

In the United States (U.S.), racial and ethnic disparities in maternal and reproductive health have persisted for centuries, and so has our collective awareness of them. Federal statistics from 1900, before the invention of the radio, indicated that death during childbirth was twice as likely for Black women as it was for white women^1^. In 1985, the Department of Health and Human Services (HHS) issued the first Report of the Task Force on Black and Minority Health, to identify and consolidate the factors that influence health status for Black, Hispanic, Asian/Pacific Islander, and Native American communities. This report was created not only to be an internal departmental resource, but “as the generating force for an accelerated national assault on the persistent health disparities”^2^. In 2000, HHS again set the goal to eliminate health disparities by 2010, addressing heart disease, obesity, and sexually transmitted infections, among others^3^. Over a century later, while technology has advanced from radio to artificial intelligence and robotics, Black women of childbearing age are suffering and dying at an even greater rate than white women. Black women die from complications of pregnancy at a rate of 3.55 times higher than white women and are now five times more likely to be diagnosed with preeclampsia, eclampsia, postpartum cardiomyopathy—the leading causes for pregnancy-related deaths^4^.

Even with decades of attention and evolving technology, these discrepancies persist due to our collective failing to resolve historically rooted systemic injustices, such as discrimination, racism, and gender and other socio-economic inequalities. Our ineptitude to acknowledge, account for, and rectify these injustices directly impacts the health and wellbeing of individuals and communities, especially women of color who contend with compounding intersection of multiple forms of oppression^5^. Oppression is the process of practices, systems, behaviors, and beliefs by which a dominant group controls, excludes, and limits access, resources, status, and power and subordinates another group to maintain privilege^6^. The result is overtly displayed in marked racial/ethnic disparities in reproductive health outcomes, healthcare access, and healthcare utilization. Healthy People 2030 defined discrimination as “a socially structured action that is unfair or unjustified and harms individuals and groups. Discrimination can be attributed to social interactions that occur to protect more powerful and privileged groups at the detriment of other groups”^7^. The report identified discrimination, both interpersonal and structural forms based on race, gender, age, disabilities and sexual orientation, and their intersectionality impacts individuals and communities but fails to cite what actions are needed to make permanent change^7^. A clear path is needed to achieve health equity, such as policies, programs, and actions that integrate a multi-level understanding of dismantling structural racism to resolve health inequities and reduce disparities. This commentary describes forms of racism at the societal, community, and individual that impact women of color over their lifespan and identifies emergent policies and actions that can guide us to an era of lasting advancement reflected in human wellness.

## Racism and reproductive health

Levels of racism have been defined as interpersonally mediated, internalized, and institutional^8^. Additionally, structural racism is how racial discrimination in its entirety is pervasively and deeply embedded and reinforced in systems, institutions, laws, written or unwritten policies, and entrenched practices and beliefs that produce, condone, and perpetuate widespread unfair treatment and oppression of people of color and lead to adverse health consequences^9^. Structural racism in the U.S. has unfairly given societal advantages to white people at the expense of people of color. According to the CDC, social determinants (i.e., education, employment, housing, transportation, and wealth) are key driving factors of health. For people of color, lack of education, employment, housing, transportation, and opportunity to accumulate generational wealth have led to poor health and life outcomes^10^. Increasingly, research focuses on structural racism and its interplay with social determinants of health to deepen our understanding of the disproportionately negative reproductive health outcomes for people of color^9,11^. A recent conceptual framework derived from Black women’s perspectives on structural racism across the reproductive lifespan identified nine domains: Housing, Medical Care, Law Enforcement, Negative Societal Views, Hidden Resources, Employment, Education, Community infrastructure, and Policing Black Families^11^. These domains are woven into discussion below to deepen our understanding of how these determinants impact health.

A stark example of structural racism in housing and its lasting legacy on reproductive racial health disparities is a policy known as redlining^9^. In 1930, this federal policy promoted segregated neighborhoods by denying loans to areas deemed too risky and increasing lending to areas considered more desirable. This policy was enacted to ensure white middle-class residents had safe, exclusive, affordable and segregated housing. Geographical maps for lenders were created designating areas by desirability from best to hazardous along racial and ethnic lines, forcing minorities to purchase in specific (more hazardous) areas^12^. This original policy was deemed illegal in 1968, when the Fair Housing Act banned racial discrimination in housing^13^. However, banks and mortgage lenders continue to use race-based punitive “redlining” practices and financial protocols that promote predatory lending, reduce mortgage options, limit credit access, and devalue home prices^14^. The result is the perpetuation of segregated neighborhoods by race and inequitable access to living in healthy neighborhoods^9^. The original policy and these current practices continue to have lasting effects on reproductive racial health disparities.

Research shows that Black and Latinx neighborhoods are associated with higher concentrations of poverty, lower-performing schools, fewer high-wage jobs, low-grade houses, dilapidated buildings and grounds, increased exposure to harmful pollutants, and inadequate essential city services, including ambulance services and reliable public transportation^15,16^. Higher rates of preterm birth, decreased gestational age, and low birth weight remain significant and can still be traced to areas once classified as hazardous compared to those known as the best neighborhoods in Los Angeles, San Francisco, and Oakland^17^.

Housing segregation has lasting implications for healthcare utilization, healthcare access, and healthcare quality at the community and individual level^9^. A lack of private sector investment in specific neighborhoods, result in a lack of employment opportunities with strong wages and job security that offer strong benefits like high-quality, consistent employer-based insurance, and a lack of high quality healthcare infrastructure to provide quality services ^9,18^. Both perpetuate a tiered healthcare system by design, based on proximity to facilities and ongoing access to reliable, comprehensive insurance, generally employer sponsored^19^. Moreover, the maldistribution of full-service quality reproductive health infrastructure and inconsistent insurance coverage with a less comprehensive scope of health benefits directly limits both pregnant and non-pregnant people’s access to high quality care. For those living in poor and under-resourced neighborhoods, it is considerably more arduous to obtain regular prenatal care and sexual and reproductive health services, obstetrics and gynecology specialist appointments, nutrition consultations, mental health services, laboratory testing and ultrasounds, or terminate a pregnancy^20^. Reproductive health service utilization for family planning and maternal health services is also affected by cost, distance, cultural and language barriers, inability to take time off work, and lack of reliable transportation or childcare. The result is a delay in care or forgoing it all together^9,21^.

In addition, community health services in Black and Latinx communities are often offered by providers who are not racially or culturally representative of the community and who do not actually reside in these communities^9^. Racially discordant provider and patient relationships are more prone to discrimination and bias exhibited through the exercise of power relationships, neglect, unjust infliction of insults, microagression or derogatory acts, that negatively impact individuals^22^. On the interpersonal and institutional level, harm, maltreatment, aggression, neglect, coercion, and abuse by providers, nurses and staff towards Black and Brown women has been well documented in labor, birth, and family planning^22–24^. Racial discrimination in reproductive health leads to misdiagnosis, lack of pain management, and ineffective care^22^.

Other domains of structural racism such as law enforcement and policing of Black families impact women’s health across the reproductive lifespan. Disproportionate levels of incarceration, increased police surveillance/interactions and police brutality are higher in communities where women of color live^9^. Recent studies have shown an association between higher odds of preterm birth for pregnant people of color who live in communities with higher police presence compared to white women who live in areas with low police presence^25^.

## Emerging policies and actions

As we noted earlier, racism is a systematic phenomenon embedded in our society’s structures, including housing, healthcare, community services, and interpersonal relationships. Solutions to address racism and the health disparities it causes must start, as Mendez and Scott posit, with the “R4P” approach: *Remove* power structures and imbalances that promote inequalities for Black women; *Repair* in a historical context the damage generated since Black people were forcibly brought to America (Understood as a whole continent from Argentina to Canada) and the impacts of denning their humanness; *Restructure* policies, systems, institutions, and norms that are continuously putting Black women under risk and cumulative stress; *Remediate* immediate needs of Black women while structural needed changes are made; and finally, *Provide* interventions that tear apart those barriers to access to resources to vulnerable, minoritized, and affected population^26^.

In order to achieve concrete actions, The R4P approach should be accompanied by McLemore’s 3R Model: *Retrofit* reproductive health services, *Reform reproductive rights*, and *Reimagine existent structures by using the reproductive justice framework*^27,28^. Together, R4P and 3R can guide policies and actions to achieve reproductive health equity, such as universal access to healthcare and insurance, not employment-based, but access to both that considers the systemic inequalities that affect Black women during their life course in changes in employment, abortion coverage, food security, householding, transportation, digital access to telehealth, and environmental conditions. Actions to decrease disparities on the federal level would include paid family leave, universal childcare, diversifying the workforce, and building an accessible and comprehensive reproductive health system with respectful racially concordant care to meet the needs of women of color through their lifespan^29,30^.

Building on this coupled approach, specific changes needed in maternal healthcare provision would go beyond considering the particular moment of pregnancy, but manifest reproductive justice throughout the life cycles^31^. To decrease racial disparities, maternal and reproductive healthcare provision services need to be fully integrated with family planning, the antenatal period, pregnancy, lactation, puerperium, pleasure, reproductive endocrinology, abortion provision, gynecologic healthcare, primary care, cardiology, and oncology. It is also necessary for the creation of more communities of care that promote racial concordance and the integration of Black midwifery, lactation support persons and doulas that address the ancestral and traditional knowledge in the healthcare system^32–40^. To achieve greater racial concordance in healthcare, changes are needed first in medical, nursing and midwifery education by increasing the number of culturally responsive training programs at low cost that provide Black and Latinx mentorship, engaged community partnerships, anti-racist curricula, and culturally supportive learning environments for aspiring students^41–43^. Infused with this deeper understanding of how to concretely confront the complexity of structural racism, more applicable policies and actions that promote health equity have emerged.

## Promising strategies to address reproductive health inequities

Visible advancements are occurring. The recent expansion of the Patient Protection and Affordable Care Act allows state Medicaid programs to extend coverage for up to 12 months postpartum^44^. This increase in access to care and coverage of services is important, as roughly 12 percent of pregnancy-related deaths occur after the first 6 weeks after birth^45^. Proposed incremental efforts on the federal level are beginning to influence a deeper understanding and consideration of above systemic inequalities for women of color at state and community levels. In an effort to reduce maternal mortality and morbidity, the 2023 presidential budget includes $470 million for expanding rural maternal health initiatives, creating pregnancy home demonstration projects, providing implicit bias training for healthcare providers, and supporting the perinatal workforce^44^. Three other such exemplars are the proposed Black Maternal Health Momnibus Act of 2021, and the executive actions to convene all Cabinet Secretaries and agency leaders to discuss a whole-of-government approach to addressing discrepancies in maternal mortality and morbidity, and the Department of Health and Human Services devoted $4.5 million dollars to train and support community doulas^44,46^.

Emerging solutions are particularly effective when community-led, both centering women and harnessing the leadership and strength of social support within communities of color. Regional examples that demonstrate how racially respectful care in reproductive health can improve outcomes include CHOICES in Memphis, TN, Kindred Space in Los Angeles, CA, Mamatoto Village in Washington, DC, and Mahogany Milk in Greensboro, NC. For instance, BeLovedBirth Black Centering in Oakland, CA offers a group prenatal model with continuity of care and racially concordant physician, midwifery and doulas and wrap around services on location, while collaborating with local partners to deliver fresh foods from farmers of color and meals from restaurants owned by people of color to women postpartum, who may need additional support for themselves and their infants at this crucial time. These responsive models are only a small representation of the change that is happening nationwide at the community and regional levels. Structural racism is overtly challenged when community birth innovations and federal policy like the expansion of postpartum services centers the leadership, the health experiences, and the lives and deaths of women of color throughout the process, from the articulation of the problem to creation of the solution.

There is no single solution to addressing systemic and structural racism or the devastating effects on reproductive health of minoritized individuals. While important recent policy gains show promise, significant work and greater financial investments at the federal and regional levels are needed to operationalize and advance the ideas laid forth in this paper. Now is the time to be bold in our policies and actions. To achieve health equity for Black women, a collective approach that incorporates the R4P and 3R Models, reproductive justice, and community expertise is needed. Together, these frameworks provide an opportunity to create lasting and sustainable change for Black women and their families.
